# Supplementary material for: COVID-19 and diet: efforts towards a balanced diet and sustainable nutrition among university students in Pakistan
Source: PeerJ. 2024 Jan 3;12:e16730. doi: 10.7717/peerj.16730 (PMC10771080; doi:10.7717/peerj.16730)
Supplement: Supplemental Information 3 [file peerj-12-16730-s003.docx]

**Appendix. 1: Nutrition and Health Information Questionnaire**

Please complete this form as completely as possible. The more information you submit, the more we will be able to adapt our time together to match your specific nutritional needs and objectives. All responses are kept private.

Name: _________________________ Student ID#: ____________________

Gender: _________________________ Age: __________________________

Height: __________________________ Weight: ________________________

# Medical/Health History

1. Please list any past or current medical conditions that you have or are currently being treated for:
2. Do you have any food allergies or medically diagnosed intolerances? Y / N (Circle one)

If yes, please list:

1. Do you take any vitamin/mineral/herbal/sports supplements? Y / N (Circle one)

If yes, please list:

1. Do you smoke? Y / N (Circle one)

If yes, how often/how much: _____________________________________

1. Please rate your daily stress level:

| 1 2 | 3 | 4 | 5 | 6 | 7 | 8 | 9 | 10 |
| --- | --- | --- | --- | --- | --- | --- | --- | --- |
| Low Stress |  |  |  |  |  |  |  | High Stress |

1. How do you cope with stress in your daily life?

# Information Regarding COVID-19

1. Do you know about immunity?

1. Not aware (b) Moderately aware (c) Fully aware
2. Do you think immunity plays a vital role in CoVID-19 infection?
3. Not aware (b) Moderately aware (c) Fully aware
4. First initial symptom of COVID-19?

(a)Not aware (b) Moderately aware (c) Fully aware

1. Eating or contacting wild animals would result in the infection by the COVID-19 virus?

(a)Not aware (b) Moderately aware (c) Fully aware

1. (COVID-19) virus spreads via respiratory droplets of infected individuals?

(a)Not aware (b) Moderately aware (c) Fully aware

1. From where you get your information about (COVID-19)?
2. News Channel (b) NCOC website (c) Seminar/Awareness sessions
3. Do you wear general medical masks to prevent the infection by the COVID-19 virus?
4. Always (b) Not at all (c) Sometimes
5. Do you use hand sensitizer after touching foreign surfaces outside your house?

(a) Always (b) Not at all (c) Sometimes

1. Do you still shake hands with people after Corona-virus pandemic? If yes, how frequently?

(a) Always (b) Not at all (c) Sometimes

# Food & Nutrition History

1. Protein is needed for the building and repair of body tissues
2. Not aware (b) Moderately aware (c) Fully aware
3. Carbohydrates provide energy for the body
4. Not aware (b) Moderately aware (c) Fully aware
5. Carbohydrates raise blood sugar levels
6. Not aware (b) Moderately aware (c) Fully aware
7. Glucose stimulates the production of insulin
8. Not aware (b) Moderately aware (c) Fully aware
9. Do you know about omega3 fatty acids?
10. Not aware (b) Moderately aware (c) Fully aware
11. Sources of vitamins and minerals are protective foods
12. Not aware (b) Moderately aware (c) Fully aware
13. Vitamin C helps to enhance immunity
14. Not aware (b) Moderately aware (c) Fully aware
15. Vitamin D helps to maintain calcium homeostasis

1. Not aware (b) Moderately aware (c) Fully aware
2. Calcium helps to maintain and build strong bones and teeth
3. Not aware (b) Moderately aware (c) Fully aware
4. Milk and other dairy products are important for bone health Water assists in removing waste products from the body
5. Not aware (b) Moderately aware (c) Fully aware
6. Water helps to maintain homeostasis in the body
7. Not aware (b) Moderately aware (c) Fully aware
8. Water transports nutrients to cells in the body
9. Not aware (b) Moderately aware (c) Fully aware
10. **Eating Practices of Male and Female Students**
11. Do you skip breakfast and having only lunch and dinner?
12. Not aware (b) Moderately aware (c) Fully aware (d) Highly disagree
13. Do you Skip lunch?
14. Not aware (b) Moderately aware (c) Fully aware (d) Highly disagree
15. Do you eat breakfast at home before going to work?
16. Not aware (b) Moderately aware (c) Fully aware (d) Highly disagree
17. Do you eat breakfast at work?
18. Not aware (b) Moderately aware (c) Fully aware (d) Highly disagree
19. Do you carry prepared breakfast from home to work?
20. Not aware, (b) Moderately aware, (c) Fully aware (d) Highly disagree
21. Do you Buy food at work from University cafe?
22. Not aware, (b) Moderately aware, (c) Fully aware (d) Highly disagree
23. Do you buy food from fast foods restaurants?
24. Not aware (b) Moderately aware (c) Fully aware (d) Highly disagree
25. Do you eat at snack time?
26. Not aware (b) Moderately aware (c) Fully aware (d) Highly disagree
27. Do you eat balanced meals three times daily?
28. Not aware (b) Moderately aware (c) Fully aware (d) Highly disagree
29. Do you buy snacks as lunch?
30. Not aware (b) Moderately aware (c) Fully aware (d) Highly disagree
31. Do you consume soft drinks daily?
32. Not aware (b) Moderately aware (c) Fully aware (d) Highly disagree
33. Do you have dinner at 6 PM daily?
34. Not aware (b) Moderately aware (c) Fully aware (d) Highly disagree
35. Do you buy breakfast or lunch from food vendors?
36. Not aware (b) Moderately aware (c) Fully aware (d) Highly disagree
37. How many times a day do you typically eat? ____________________________

`15. Do you consume caffeinated beverages on a regular basis? (Check all that apply)

__________ Coffee ____________ Drink____________ Soda _____________ Energy Drink

1. Do you avoid any of the following foods? (Check all that apply)

| Red meat | Fruits | Sweets (candy, desserts) |
| --- | --- | --- |
| Poultry (chicken, turkey) | Fried food | Alcohol |
| Fish | Breads | Fats/oils (mayo, dressing, butter) |
| Dairy (milk, cheese) | Grains (pasta, rice) |  |
| Vegetables | Fast food |  |

1. Foods you especially like:
2. Foods you especially dislike:

# Weight History

1. Has your appetite changed recently? Y / N (Circle one)

If yes, please describe:

1. Have you recently gained or lost weight? If yes, please explain whether it was a gain or loss and what changes led to the change in weight.
2. Have you ever had concerns about your weight? Y / N (Circle one)

(a)Over weight (b) Underweight

Comment:

1. Have you ever tried to lose or gain weight in the past? Y/N (Circle one)

If yes, please describe:

1. Overall, how satisfied are you with the physical appearance of your body?
2. Very satisfied (b) Somewhat dissatisfied (c) Somewhat satisfied (d) very dissatisfied

# Physical Activity History

Are you currently physically active? Y / N (Circle one)

If yes, how often: _________ timesperweek.

How long: minutes per session.

Type of activities:

Please rate the average intensity of your workouts:

Light (walking slowly, sitting, standing)

Moderate (walking briskly, heavy cleaning and light bicycling)

Vigorous (hiking, running, fast bicycling, most team sports, weight lifting)

# Nutrition Goals

1. What nutrition-related goals do you have? What eating habits would you like to work on?
2. How important is it to you to make changes in your nutrition habits? (Please circle)

1 2 3 4 5 6 7 8 9 10

Unimportant Very Important

1. How confident are you in your ability to improve your nutrition habits? (Please circle)

1 2 3 4 5 6 7 8 9 10

Unimportant Very Important

Can we publish your data for research purpose Y / N (Circle one)
